# Modeling and Optimization of *Lactobacillus sakei* Application and Vacuum Tumbling for Improving Functional and Technological Properties of Cull Cow Beef

**DOI:** 10.3390/foods15122063

**Published:** 2026-06-07

**Authors:** Aigul Tayeva, Berdikul Rskeldiyev, Nazym Abilmazhinova, Mamura Absalimova, Maria Momchilova, Elena Ushanskaya, Dariya Tapalova, Akmaral Kurmanbekova, Aiman Smagulova

**Affiliations:** 1 Department of Food Technology, Almaty Technological University, 100 Tole bi Str., Almaty 050012, Kazakhstan; aigul.taeva@gmail.com (A.T.); berdan_r@mail.ru (B.R.); dariyaxt@gmail.com (D.T.); akma3108@mail.ru (A.K.); s.aiman76@mail.ru (A.S.); 2 Institute of Preservation and Quality, Agricultural Academy, Mendeleev 12, 4000 Plovdiv, Bulgaria; masha821982@abv.bg; 3 Department of Nutrition Named After Academician T. Sharmanov, Asfendiyarov Kazakh National Medical University, 94 Tole bi Str., Almaty 050012, Kazakhstan; ushanskaya.e@kaznmu.kz

**Keywords:** *Lactobacillus sakei*, tenderization, meat, mathematical modelling

## Abstract

Meat from culled cows is characterized by increased toughness and limited processing suitability, yet the combined effects of microbial cultures and mechanical processing have not been sufficiently studied. The aim of this study was to evaluate the effect of *Lactobacillus sakei* (SafePro B-2, 10^11^ CFU/g) in combination with vacuum massage on the physicochemical, structural, and functional–technological properties of culled cow meat, as well as to optimize the process using response surface methodology. It was found that the combined treatment led to a 25–35% reduction in shear force and a 12–18% increase in water-holding capacity compared to control samples. The pH value decreased from 6.2 to 5.6–5.8, indicating active lactic acid fermentation. Modeling using the response surface method demonstrated high predictive power of the models (R^2^ = 0.91–0.96) and allowed for the determination of optimal process parameters (10^7^ CFU/g; 40 min of mixing), ensuring maximum softening and product stability. Analysis of the texture profile revealed a statistically significant (*p* < 0.05) decrease in hardness, stickiness, and chewiness, accompanied by an increase in cohesiveness, indicating a reorganization of the protein matrix at the microstructural level. Additionally, microstructural modifications and reorganization of the protein matrix were observed, accompanied by changes in amino acid composition, indicating a change in the state of proteins and their spatial distribution within the meat matrix. Overall, the combined use of *Lactobacillus sakei* and vacuum massaging effectively improves the functional, technological, and structural characteristics of low-grade beef raw materials. The proposed approach can be considered a scientifically sound strategy for optimizing processing parameters and increasing the efficiency of utilizing meat from culled cows.

## 1. Introduction

Meat obtained from culled cows is characterized by reduced functional and technological properties, primarily due to increased toughness associated with age-related changes in muscle tissue [1]. With advancing age, the proportion of connective tissue proteins, such as collagen and elastin, increases, alongside significant alterations in their structural organization. In particular, the accumulation of thermostable intermolecular cross-links (e.g., pyridinoline and deoxypyridinoline) reduces collagen solubility and limits its susceptibility to thermal denaturation [2]. Although the total collagen content in older animals may be comparable to that in younger ones, its solubility is substantially lower (~5.5% vs. ~10.5%), contributing directly to increased meat toughness [3].

Simultaneously, age-related modifications occur in myofibrillar proteins, including decreased activity of endogenous proteolytic systems (e.g., calpains), increased inhibition by calpastatin, and accumulation of oxidative modifications [4]. These factors restrict post-mortem proteolysis of key structural proteins such as desmin, titin, and nebulin, resulting in limited myofibril weakening during maturation. Consequently, meat from culled cows exhibits higher shear force values, reduced tenderness, and pronounced structural heterogeneity [5]. These characteristics highlight the need for targeted tenderization strategies specifically adapted to this type of raw material.

Various technological approaches have been developed to improve the quality of tough meat, including mechanical treatment, marination, enzymatic tenderization, and the use of microbial cultures [6]. Mechanical methods, such as tumbling and massaging, enhance brine distribution and protein extraction, whereas marination—particularly under acidic conditions—can weaken connective tissue structures and improve tenderness [7]. At the same time, starter cultures are widely used, primarily lactic acid bacteria (LAB), which help to optimize the physical and technological properties. In particular, there is a reduction in firmness and shear force, an increase in tenderness and juiciness, and an improvement in water-binding capacity, as well as stabilization and intensification of color [8]. Despite the availability of these methods, their effectiveness is often inconsistent and highly dependent on raw material characteristics and processing conditions. In particular, their application to meat from culled cows remains insufficiently optimized, and the combined effects of different technological factors are not fully understood.

Among LAB, *Lactobacillus sakei* (*L. sakei*) is widely recognized as a key species in meat fermentation due to its adaptability to meat systems, its antagonistic activity and its ability to rapidly acidify the medium [9,10]. It has previously been shown that *L. sakei* is capable of participating in protein modification, including proteolysis and structural changes in muscle proteins, which may affect texture and water-binding capacity [11,12]. Thus, the results of earlier studies showed that *L. sakei* strains can be used in the production of dry fermented sausages, as they improve the technological characteristics. It was found that the effect of *L. sakei* on fermented sausages led to favorable changes in the textural characteristics of the fermented sausages [13,14].

Vacuum massaging is a widely used process designed to improve brine distribution, accelerate mass transfer and increase the degree of hydration of muscle proteins [15,16]. It also promotes structural loosening of muscle tissue [17,18]. The combination of *L. sakei* application with vacuum massaging represents a potentially synergistic approach: mechanical action may facilitate the penetration and uniform distribution of microbial cells and metabolites, while also enhancing the manifestation of their proteolytic and acidifying effects [19,20]. Despite this, the combined influence of these factors on the structural and functional properties of meat, particularly from culled cows, has not been systematically investigated.

Several studies have demonstrated that vacuum massaging significantly influences the functional and technological properties of meat systems, particularly by improving water-holding capacity and reducing processing losses [21,22]. It has also been reported that vacuum conditions contribute to a more uniform distribution of curing solutions within muscle tissue, resulting in more consistent physicochemical characteristics [23]. In addition, researchers have shown that massaging leads to partial disruption of muscle fiber structure and weakening of connective tissue components, which is associated with a decrease in shear force and improved tenderness [24]. The effectiveness of this process is strongly dependent on technological parameters such as rotation speed, processing time, and vacuum level, which determine the intensity of structural modification [25].

Despite extensive research into the individual effects of lactic acid bacteria (*L. sakei*) and mechanical processing, there are virtually no comprehensive studies on their combined use to improve the functional and technological properties of beef from culled cows. Furthermore, the use of quantitative models to describe these processes and optimize technological parameters is limited in the literature.

Thus, the aim of this study is to investigate the combined effect of adding *L. sakei* to the brine and applying vacuum massaging on the physicochemical, structural–mechanical, color and microstructural characteristics of meat from culled cows, as well as the development of predictive models using the response surface method (RSM) and multi-response optimization for the quantitative description of tenderization processes and the determination of optimal technological regimes.

## 2. Materials and Methods

### 2.1. Materials

Raw meat sample products were prepared from muscle tissue of the hip cut. To ensure anatomical homogeneity, samples were taken from the muscle tissue of the hip region (skeletal muscles of the thigh), which share comparable physiological functions and structural properties. Prior to further processing, visible connective tissue, tendons and excess subcutaneous fat were carefully removed to minimize variability associated with morphological heterogeneity of the source material. The meat of culled cows of the Holstein breed, 6–7 years old, purchased from the peasant farm “Amantai” (Almaty, Kazakhstan), was used as the main raw material.

*L. sakei,* strain SafePro^®^ B-2 (Chr. Hansen, Horsholm, Denmark), with a titer of 10^11^ CFU/g, was used as a starter culture; the dosage was based on the titer stated by the manufacturer and the standardized starter culture dosage used in the preparation of the brine.

Vacuum tenderization of raw meat was carried out on the ETDU unit equipment, model TUZ. The equipment was developed according to an individual technical specification for the needs of Almaty Technological University (Almaty, Kazakhstan) and is used for experimental purposes in the development of new meat products.

The phosphate mixture was Belfos-90 (manufacturer: Belprodukt CJSC, Gomel, Belarus). The mixture contains sodium pyrophosphates and triphosphates and is designed to improve the functional and technological properties of minced meat.

For salting, a nitrite curing mixture (0.6% NaNO_2_ and 99.4% NaCl) was used, manufactured by Damu-Khimiya LLC (Karaganda, Kazakhstan), 25 kg packaging. The mixture meets the requirements of TR CU 029/2012 and is used to stabilize color, inhibit pathogenic microflora, and extend the shelf life of meat products.

Prior to conducting the experimental procedures, quality control was carried out on the raw meat. Beef was used, sourced from culled adult dairy cows from a single production source, which ensured the homogeneity of the batch. The freshness of the meat was assessed based on organoleptic indicators (color, odor, surface texture). All samples approved for further use in the experiments showed no signs of spoilage or off-odors. Physical and chemical parameters were additionally monitored, including the pH of muscle tissue, which was within the range typical of normal post-mortem meat maturation, thereby allowing the exclusion of raw material showing signs of PSE or DFD. The technological properties of the meat affecting processing and the quality of the final product were also taken into account, in particular the structure of the muscle tissue (visual assessment during boning and trimming).

Prior to sample preparation, the meat underwent standard trimming to remove visible connective tissue, tendons and excess fat, thereby minimizing variability and eliminating the influence of initial factors on the experimental results.

The study used meat and a starter culture of *L. sakei*, which was added to the brine solution used for injection. The brine was prepared according to a standard process, followed by massaging of the meat. All reagents and materials were food-grade.

To clarify the experimental structure and avoid pseudo replication, biological and technical replication were separated at both the experimental and statistical stages. Meat samples obtained from five culled Holstein cows were considered biological replicates and were treated as independent experimental blocks. From each animal, anatomically comparable portions of the hip muscle were prepared, trimmed under the same conditions and randomly assigned to the nine factorial treatments and the untreated control group. Random allocation was used to reduce systematic bias associated with intramuscular heterogeneity, while the order of sample processing and subsequent instrumental measurements was randomized within each analytical session. Technical replicates represented repeated measurements performed on the same biological sample and were used to estimate analytical repeatability. They were not treated as independent biological observations in inferential statistics. For statistical analysis, technical replicates were averaged within each animal × treatment combination, whereas animal identity was considered a biological blocking factor. Since all raw material originated from a single production source and one controlled raw-material lot, between-batch variability was minimized; however, the limited number of raw-material batches was considered a methodological limitation when interpreting the generalizability of the results.

### 2.2. Sample Preparation

The study used boneless beef obtained from culled adult dairy cows. The meat was pre-treated and then cut into portions weighing 0.4 kg.

To ensure the homogeneity and comparability of the samples, selection was carried out from anatomically identical cuts obtained from each animal (*n* = 5), represented by skeletal muscles with similar morphology and functional load. Standardized samples were prepared from each cut. During sampling, muscles that differed significantly in muscle fiber type and connective tissue content were excluded. Samples were taken from different regions of each cut and subsequently randomized, which increased the representativeness of the samples and reduced intramuscular variability.

The brine was injected at a rate of 15% of the raw material’s weight, resulting in a final salt content in the product of 1.875% (≈2%), which is considered optimal for meat products. The control brine had the following composition: 12.5% table salt, 2.5% sucrose, 0.07% sodium nitrite, 1.5% phosphates and 83.43% drinking water. The brines for experimental samples were prepared on the same basis, but *L. sakei* was additionally incorporated according to the experimental design, whilst the amount of drinking water was adjusted accordingly to maintain the total brine mass.

After preparation, the samples were randomly allocated to the experimental groups, thereby eliminating systematic sampling bias and ensuring the statistical validity of the results.

After injection, the samples were held for 18 h at 4 °C to allow equilibration and uniform distribution of curing ingredients, in accordance with previously reported curing and marination methods for meat systems [26,27,28].

The samples were then transferred to a vacuum tenderizer and massaged at 20 rpm for the time specified by the experimental design.

**Figure 1:**
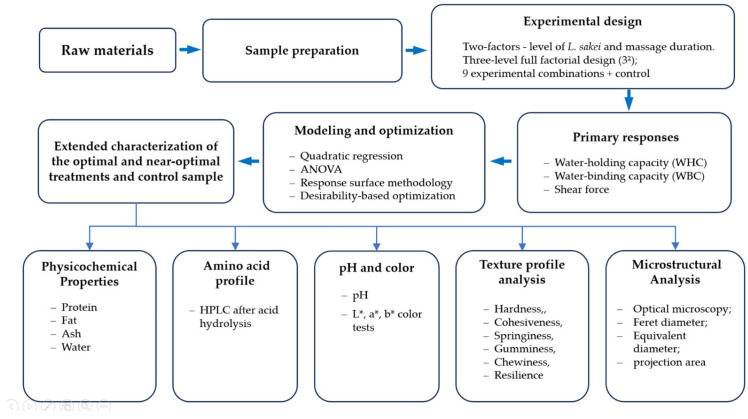
Experimental procedure.

To provide a clear overview of the experimental design and the sequence of the study stages, a flowchart of the experiment was developed (Figure 1). The diagram illustrates the main stages of the study, including the preparation of meat raw materials, the formation of samples, their allocation to experimental groups, brine injection, ageing, and the subsequent physical–chemical, textural and microstructural analyses.

### 2.3. Mathematical Modeling and Optimization of Process Parameters

The study was carried out using a two-factor, three-level full factorial design (3^2^), which included 9 experimental combinations and control sample. The 9 experimental combinations represented the full set of treatment conditions obtained by combining each level of *L. sakei* addition with each level of massage duration, as presented in Table 1. A control sample was prepared separately from the same raw material without the addition of *L. sakei* and without massage treatment and was used only as a baseline reference. The independent variables were the level of *L. sakei* added relative to raw material mass and massage duration.

The factor ranges were selected to represent a biologically relevant and technologically feasible short-time microbial–mechanical tenderization domain rather than arbitrary experimental limits. The selected *L. sakei* levels of 0.005–0.015% corresponded, according to the declared culture titer of 10^11^ CFU/g, to approximately 5 × 10^6^–1.5 × 10^7^ CFU/g of raw meat. This range covers the starter-culture level commonly used in meat fermentation and meat bio protection studies, while remaining below very high inoculation levels, such as 10^8^ CFU/g, which are mainly applied in controlled dry-fermented sausage systems rather than in injected non-fermented beef matrices [29]. The tumbling duration range of 20–60 min was selected as a short-time mechanical treatment interval. The lower level of 20 min was used to evaluate the initial redistribution of brine and microbial cells, the central level of 40 min represented an intermediate processing condition, and the upper level of 60 min represented an intensive but still moderate short-time tumbling treatment. Previous studies have shown that vacuum tumbling and tumbling-curing can improve tenderness, water-holding capacity, cooking yield, myofibrillar fragmentation, protein hydration and structural properties of meat systems [30]. Therefore, the two-factor, three-level full factorial design was considered appropriate for estimating linear, quadratic and interaction effects of microbial dose and tumbling duration within the predefined experimental domain [31].

The response variables used to assess the process were water-holding capacity (Y_1_, WHC, %), water-binding capacity (Y_2_, WBC, %), and shear force (Y_3_, N).

The mathematical treatment of the experimental data was performed in STATISTICA 13 (TIBCO Software Inc., Palo Alto, CA, USA). Only the experimental samples of the three-level full factorial design were used for model fitting and optimization, whereas the control sample was used only as a baseline reference for comparative interpretation.

Prior to model development, the experimental dataset was preliminarily characterized using descriptive statistics, including the arithmetic mean, standard error, median, standard deviation, variance, skewness, kurtosis, coefficient of variation, and minimum and maximum values of the response variables. This step was used to evaluate the general distribution pattern of the experimental data and the suitability of the dataset for subsequent regression analysis.

Because the effects of the studied factors were expected to be nonlinear within the selected experimental domain, the response variables were described using a full second-order polynomial regression model. The model used for each response had the following general form:

(1) $$Y_{i}=\beta_{0i}+\beta_{1i}x_{1}+\beta_{2i}x_{2}+\beta_{12i}x_{1}x_{2}+\beta_{11i}x_{1}^{2}+\beta_{22i}x_{2}^{2}$$

where Y_i_ is the predicted response; i = 1,2,3 corresponds to Y_1_–Y_3_; x_1_ is the coded value of SafePro B-2 content relative to raw material mass; x_2_ is the coded value of massage duration; β are the regression coefficients estimated by the ordinary least squares method.

The statistical significance of the regression coefficients was evaluated using Student’s *t*-test and the corresponding *p*-values, and 95% confidence intervals were determined for all model terms. Model adequacy was assessed by analysis of variance (ANOVA) using Fisher’s F-test, as well as by the multiple correlation coefficient (R), the coefficient of determination (R^2^), the adjusted coefficient of determination (adjusted R^2^), and the standard error of estimate. In addition, the Durbin–Watson statistic was calculated to evaluate the presence of serial correlation in the residuals [32].

Multi-response optimization was performed using the desirability function approach implemented in STATISTICA 13 as Response desirability Profiling. The optimization problem was formulated within the experimental domain as follows:

$$Y_{1}=f_{1}(x_{1},x_{2})\to max$$

$$Y_{2}=f_{2}(x_{1},x_{2})\to max$$

$$Y_{3}=f_{3}(x_{1},x_{2})\to min$$

The individual desirability functions for the three responses were combined into an overall desirability index according to the Derringer–Suich approach:

(2) $$D=(d_{1}\cdot d_{2}\cdot d_{3}{)}^{1/3}\to max$$

where d_1_–d_3_ are the individual desirability values for Y_1_–Y_3_, respectively, ranging from 0 to 1. In the optimization procedure, WHC (Y_1_) and WBC (Y_2_) were targeted for maximization, whereas shear force (Y_3_) was targeted for minimization. The factor combination corresponding to the highest composite desirability value was taken as the optimal processing condition.

To visualize the effects of the factors and to interpret the fitted models, response surface plots and contour plots were generated for all response variables. The predictive performance of the obtained equations was further evaluated by comparing the experimental and calculated response values within the design space.

### 2.4. pH and Color Measurements of the Meat Samples

The pH of the meat samples was measured using a Hanna HI99163 portable pH meter (Hanna Instruments, Smithfield, RI, USA), equipped with a special meat probe (FCO99). The device was pre-calibrated using certified buffer solutions with pH values of 4.04 and 6.86.

pH measurements were taken using a penetrating electrode within the muscle tissue (in the inner, central region of the muscle), which minimized the influence of surface factors. In each sample, at least three measurements were taken at different points within the muscle tissue, and the results were then averaged.

The instrumental color of the meat samples was determined using a Konica Minolta CR-410 colorimeter (Konica Minolta Holding, Inc., New York, NY, USA), equipped with a 50 mm aperture, a D65 light source, and a standard 2° observer. The colorimeter was calibrated using a standard white plate. The values for lightness (L*), redness (a*), and yellowness (b*) were measured at three different locations on the surfaces of the meat slices exposed to oxygen [33].

Color determination was carried out on a fresh cross-section of the sample. Prior to measurement, the samples were left in contact with atmospheric oxygen for 30 min to stabilize the color (myoglobin oxygenation). Measurements were taken under standard lighting conditions at several points on the cross-section, followed by the calculation of average values.

pH and color measurements were carried out after completion of the processing (aging for control samples, vacuum kneading for experimental samples).

For pH and color measurements, instrumental validation was performed before each analytical session. The pH meter was calibrated using certified buffer solutions, and measurement stability was checked before analysis. Color measurements were carried out after calibration with the manufacturer’s standard white plate. Instrumental repeatability was assessed using repeated measurements at different points of the same sample. For each parameter, repeatability was expressed as standard deviation and relative standard deviation, while measurement uncertainty was estimated from calibration, instrumental repeatability and within-sample variability.

### 2.5. Physicochemical Properties

Determination of the mass fraction of fat was carried out by extraction method using n-hexane (analytical grade, Merck KGaA, Darmstadt, Germany), in accordance with ISO 1443:1973 [34], “Meat and meat products—Determination of total fat content.” Determination of the mass fraction of protein was carried out by the Kjeldahl method using a conversion factor of 6.25, in accordance with ISO 937:2023 [35], “Meat and meat products—Determination of nitrogen content—Reference method”.

Determination of the mass fraction of ash was carried out by drying, charring, and ash removal at a temperature of (550 + 25) °C of the test sample, followed by determination of the mass fraction of total ash., in accordance with ISO 936:1998, “Meat and meat products—Determination of total ash [36]”.

All measurements were performed in triplicate, followed by calculation of the mean value and standard deviation.

Water-holding capacity (WHC) was determined using the Grau–Hamm pressing method by measuring the area of released moisture under a standardized load.

Water-binding capacity (WBC) was evaluated by centrifugation (OPn-3M, Dastan JSC, Bishkek, Kyrgyzstan), calculating the amount of water retained in the sample after applying centrifugal force.

The physicochemical parameters were determined on independent samples for each experimental treatment *(n* = 3).

The validation of physicochemical measurements was based on calibration control, replicate analysis and uncertainty estimation. For ISO-based compositional analyses, analytical quality was controlled through repeated measurements, reagent blanks where applicable and verification of equipment calibration. WHC and WBC determinations were standardized by controlling sample mass, pressing or centrifugation conditions, temperature and measurement time. Measurement uncertainty was estimated from technical repeatability and instrument-related sources and was considered when interpreting differences between treatments.

### 2.6. Texture Profile Analysis

The texture profile of the meat samples was evaluated using the Lamy Rheology TX-700 texture analyzer (Lamy Rheology Instruments, Champagne at Mont d’Or France). The samples were cut into standard-shaped cubes with controlled orientation relative to the muscle fibers. The procedure was initiated once the equipment was properly set up. Both graphical and digital readings were obtained. The texture analyzer needle was allowed to penetrate the sample at an average speed of 80 mm per minute, perpendicular to the muscle fibers. The measured variables were the hardness, adhesiveness, cohesiveness, chewiness, gumminess and resilience of the meat samples. All measurements were performed in triplicate, followed by calculation of the mean value and standard deviation.

This device was also used to measure the shear force in meat samples according to the method [37].

For textural measurements, the texture analyzer was calibrated for force and distance before each analytical session. Sample geometry, fiber orientation, test speed and probe position were standardized for all measurements. Measurements were performed perpendicular to the muscle fiber direction, and visibly deformed or non-uniform samples were excluded from analysis. Repeatability was assessed using technical replicates, while measurement uncertainty was estimated from instrumental repeatability and with-in-sample variability.

### 2.7. Amino Acid Contents

The amino acid profile of the raw meat samples was analyzed according to AOAC Official Methods 2018.06 by the method described in [38]. The method is based on acid hydrolysis of protein followed by determination of amino acids by HPLC (high-performance liquid chromatography) after prior derivatization.

The analysis was performed on an Agilent 1260 Infinity II LC liquid chromatograph (Agilent Technologies, Santa Clara, CA, USA) with fluorescence and UV detectors. Amino acids were separated on a reverse-calibrated C18 column with gradient flow of mobile phase (acetonitrile (HPLC grade, Merck, Darmstadt, Germany): water with buffer, pH 6.5). Column temperature was 30 °C, sample volume was 20 µL, and detection wavelength was λ = 338 nm (for OPA derivatives).

Amino acids were identified by retention time of standards (Merck KGaA, Darmstadt, Germany). Quantification was performed by the external standard method using a calibration curve.

Measurements were performed in triplicate, followed by calculation of the mean value and standard deviation.

### 2.8. Determination of the Size of Structural Elements in Meat and Meat Products Using Optical Microscopy

The dimensions of meat structural elements (muscle fibers, fat inclusions, and meat particles) were determined using a Euromex Delphi-X Observer trinocular optical microscope equipped with a VC.3045-HDS digital camera (Euromex Microscopen B.V., Arnhem, The Netherlands). Image capture and subsequent morphometric analysis were performed using ImageFocus Alpha Full software package, version 1.3.7.31026 (260322).

Before starting the measurements, the “microscope–camera” system was calibrated using a certified micrometric scale. Calibration was performed at a 20× magnification, measuring a known 100 μm section of the scale in the software and then entering its actual value. The resulting calibration was saved separately for this magnification.

The samples were prepared as follows. Thin sections 10–30 μm thick were cut from the target muscle tissue using a sharp blade. The sections were placed on a microscope slide and mounted using saline solution, then covered with a cover slip. Excess fluid was removed with filter paper to prevent displacement of structural elements.

Microscopy was performed at 20× magnification in bright-field mode. Microphotographs were obtained from several randomly selected fields of view of each specimen to ensure representativeness. For analysis, areas were selected where structural elements were clearly visualized, did not overlap, and were not deformed.

The dimensions of structural elements were measured using the morphometric analysis tools of the software, including the determination of the Feret diameter, projection area, and equivalent diameter [39]. For each sample, between 30 and 200 particles were analyzed, depending on the heterogeneity of the structure. Particles with overlap, distorted shape, or insufficient focus were excluded from the analysis. The minimum and maximum dimensions of each element were recorded automatically, and the results were expressed in micrometers based on the calibration performed. Measurements were performed in triplicate, followed by calculation of the mean value and standard deviation.

### 2.9. Statistical Analysis

Statistical analysis was performed in accordance with the experimental design and the type of response variables. Response surface modelling and optimization were carried out using STATISTICA 13.0 (TIBCO Software Inc., Palo Alto, CA, USA), whereas group comparisons for physicochemical, color, amino acid, textural and microstructural data were performed using SPSS 24.0 (SPSS Inc., Chicago, IL, USA). All experimental results were obtained in triplicate and are presented as mean ± standard deviation.

The effects of *L. sakei* concentration, tumbling duration and their interaction on water-holding capacity, water-binding capacity and shear force were evaluated using a second-order polynomial regression model. Regression coefficients were estimated by the least-squares method, and their significance was assessed using Student’s *t*-test. Model adequacy was evaluated by analysis of variance, Fisher’s F-test, coefficient of determination, adjusted coefficient of determination, standard error of estimate, Durbin–Watson statistic and comparison between experimental and predicted values.

Multi-response optimization was performed using the desirability function approach.

For comparisons among control and treated samples in physicochemical, color, textural and microstructural analyses, one-way analysis of variance was applied. When significant differences were detected, Duncan’s multiple range test was used to compare group means at *p* < 0.05. Where the main objective was comparison of treated groups with the untreated control, planned control-based comparisons were additionally considered appropriate, following the logic of Dunnett-type comparisons. Multicollinearty among regression terms was checked using correlation diagnostics and variance inflation factors, since collinearity can affect the stability and interpretation of regression coefficient.

## 3. Results and Discussion

### 3.1. Experimental Design and Results of the Full Factorial Experiment

A two-factor, three-level full factorial design was used to evaluate the effects of *L. sakei* content and massage duration on the functional and textural properties of beef samples. The experimental design matrix and results for FFE experiments are shown in Table 2.

The changes observed in the factorial design can be interpreted as a combined microbial–mechanical modification of the muscle protein matrix. Compared with the untreated control, the treated samples showed higher WHC and WBC and lower shear force, indicating improved water retention and reduced structural resistance of the meat tissue. From a mechanistic point of view, water retention and tenderness in meat are mainly governed by the spatial organization, hydration and integrity of myofibrillar proteins, particularly myosin, actin and actomyosin, as well as by the interaction between myofibrillar and connective tissue structures [40]. *L. sakei* is well adapted to meat environments and can use peptides and amino acids derived from sarcoplasmic and myofibrillar proteins; transcriptomic studies have shown that, in the presence of meat proteins, *L. sakei* up-regulates genes encoding oligopeptide transporters and intracellular peptidases, which supports its involvement in protein-derived nutrient metabolism [41]. Tumbling, in turn, promotes brine penetration, salt/phosphate-assisted swelling of myofibrillar proteins, extraction of salt-soluble proteins and partial mechanical weakening of muscle fibers [42].

Before fitting the regression models, the experimental data obtained for the response variables were preliminarily characterized using descriptive statistics. The main statistical characteristics of Y_1_, Y_2_, and Y_3_ are summarized in Table 3.

Descriptive statistical characteristics provide a quantitative overview of the empirical data (regarding the mean, its variance—dispersion, and skewness) and, as a first approximation, test the assumptions underlying the regression analysis [43]. The results showed low variability of the experimental data, as indicated by standard errors below 3% of the corresponding mean values and coefficients of variation ranging from 1.2344% to 7.7874%. The mean and median values were close for all response variables, suggesting the absence of pronounced asymmetry in the dataset. The minimum and maximum values were distributed relatively symmetrically around the corresponding mean values, indicating no pronounced distortion in the data structure [44]. These characteristics indicate that the experimental data were sufficiently consistent for subsequent regression modeling.

### 3.2. Development and Statistical Evaluation of Quadratic Regression Models

To quantify the effects of *L. sakei* content and massage duration on the studied responses, second-order polynomial regression models were fitted for WHC (Y_1_), WBC (Y_2_), and shear force (Y_3_). The fitted coefficients, together with their standard errors, Student’s t-values, *p*-values, and 95% confidence intervals, are presented in Table 4.

Table 4 shows that the linear effects of both factors were statistically significant for all three response variables, indicating that the *L. sakei* level and massage duration had a pronounced influence on WHC, WBC, and shear force within the studied experimental domain. For WHC (Y_1_), both linear terms, both quadratic terms, and the interaction term were significant (*p* < 0.05), suggesting a clearly expressed nonlinear response surface. For WBC (Y_2_), the linear terms were significant, whereas the quadratic and interaction terms were not statistically significant at the selected significance level, indicating that the response was predominantly governed by the main effects of the factors. For shear force (Y_3_), both linear terms and the interaction term were significant, while the quadratic effects were not significant, indicating that the decrease in shear force was primarily associated with the direct effects of the factors, with an additional contribution from their interaction within the studied region.

The relatively large coefficients and standard errors for X_1_-related terms reflect the narrow natural range of *L. sakei* content (0.005–0.015%) and the scale dependence of the model estimates.

In addition to statistical significance, the practical relevance of the regression effects was evaluated by considering the magnitude and technological direction of the responses. Relative to the untreated control, the experimental treatments increased WHC and WBC and reduced shear force, which are technologically meaningful changes for improving the processing suitability and tenderness of meat from culled cows. The regression coefficients should not be interpreted only through their *p*-values, but also through their effect direction and their contribution to water retention and mechanical weakening of the meat matrix. The large coefficients associated with *L. sakei* concentration are partly attributable to the narrow natural scale of this factor. Therefore, coefficient magnitude alone was not used as the sole criterion of factor importance. To avoid overinterpretation of individual regression terms, Multicollinearity among the polynomial model terms was evaluated using the variance inflation factor (VIF). Since polynomial regression terms are sensitive to scaling and centering, VIF diagnostics were performed using the coded variables of the factorial design. The VIF values for the linear terms, quadratic terms and interaction term were all equal to 1000, indicating the absence of multicollinearity among the model predictors. The statistical significance and direction of the regression coefficients were interpreted together with the response surface behavior and technological relevance of the responses.

Thus, using the estimates of the *b*-coefficients, the following equations for the quadratic regression of the two brine parameters can be written as:

(3) Y_1_ (WHC, %): 58.3845 + 778.09·x_1_ + 0.068_1_·x_2_ − 0.819·x_1_·x_2_ −29,432.91·x_1_^2^ − 0.000677·x_2_^2^; R^2^ = 0.999

(4) Y_2_ (WBC, %): 61.512 + 564.312·x_1_ + 0.118·x_2_ − 3.395·x_1_·x_2_ – 13,668.3544·x1^2^ − 0.000554·x_2_^2^; R^2^ = 0.987

(5) Y_3_ (Shear force): 21.7815 − 422.7131·x_1_ − 0.0682·x_2_ + 2.3354·x_1_·x_2_ + 3769.62·x_1_^2^ + 0.00011·x_2_^2^; R^2^ = 0.996

The obtained regression models showed that increasing the *L. sakei* level and massage duration improved the functional and structural-mechanical properties of the meat system within the studied range. WHC exhibited a pronounced nonlinear response with the formation of an optimal region inside the experimental domain, whereas WBC changed more steadily and was mainly determined by the main effects of the factors. At the same time, shear force decreased with increasing factor levels, confirming the tenderizing effect of the treatment. The close agreement between the model intercepts and the observed control values additionally supports the consistency of the obtained regression equations with the baseline characteristics of the untreated samples.

To assess the degree of influence of each regression term and the quality of the second-order equation calculated from experimental data, the results of the analysis of variance were used (Table 5).

Table 5 shows that the estimated regression equation provides a good fit to the experimental data, as the sum of squares due to regression (SS_R_) accounts for a significant proportion of the total sum of squares (SS_T_) and is given by:

for Y_1_—WHC, %

(6) $$\frac{SS_{R}}{SS_{T}}\cdot 100\%=\frac{32.0376}{32.04344}\cdot 100\%=99.98\%$$

for Y_2_—WBC, %

(7) $$\frac{SS_{R}}{SS_{T}}\cdot 100\%=\frac{45.03963}{45.58945}\cdot 100\%=98.79\%$$

for Y_3_—Shear force, N

(8) $$\frac{SS_{R}}{SS_{T}}\cdot 100\%=\frac{39.0064}{39.14145}\cdot 100\%=99.65\%$$

which indicates a statistically significant (*p* < 0.05) difference between the mean squares.

To assess the quality of the regression Equations (3)–(5), the multiple correlation coefficient *R*, the coefficient of determination *R^2^*, Fisher’s *F*-test and the Durbin–Watson statistic *d* were calculated (Table 6).

The values of the statistical criteria given in Table 6 indicate that the regression equations obtained reliably and adequately describe the influence of the parameters under investigation, Y_1_, Y_2_ and Y_3_, with a 95% confidence level.

The relatively high values of the multiple correlation coefficient (R_1,2,3_ = 0.999; 0.994; 0.9983) indicate a very strong relationship between the resulting indicators Y_1_, Y_2_ and Y_3_ and the controlled variables Y_1_, Y_2_ and Y_3_ included in the study. The coefficient of determination (R_1,2,3_ = 0.999; 0.987; 0.996) accounts for 99.96%, 97.29% and 99.22% of the total variation in the corresponding response in the experimental data.

The values of Fisher’s F-test statistic, equal to 4388.712; 65.5336; 231.0635 for Y_1_, Y_2_ and Y_3_ respectively, and the calculated significance levels *p* < 0.1 indicate a sufficiently high approximating ability of the obtained equations.

As indicated by the values of the Durbin–Watson statistic d, it can be concluded that there is no serial correlation. Values of the Durbin–Watson statistic: dY_1_ = 1.7946, dY_2_ = 1.7032, dY_3_ = 1.8284. With the sample size and design of experiment, no signs of serial correlation were detected. The regression equations obtained adequately described the process in the studied domain.

Although the obtained R^2^ and adjusted R^2^ values indicate a close fit between the experimental and predicted responses, these values were interpreted cautiously because the models were developed using a limited 3^2^ factorial dataset. In small response surface designs, very high R^2^ values may reflect not only a strong process response, but also the restricted experimental domain and low residual variance. The obtained equations should be regarded as empirical interpolation models valid only within the investigated factor ranges. Additional independent validation experiments at intermediate factor combinations or using a separate raw-material batch are required before scale-up.

### 3.3. Response Surface Analysis and Multi-Response Optimization

**Figure 2:**
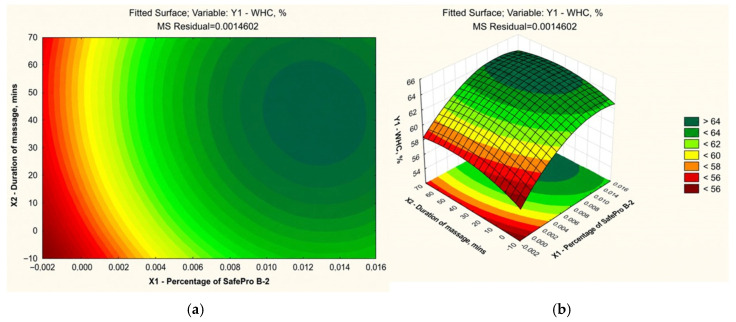
(**a**) Response surface for Y_1_ and (x_1_ and x_2_); (**b**) Contour lines for Y_1_ and (x_1_ and x_2_).

**Figure 3:**
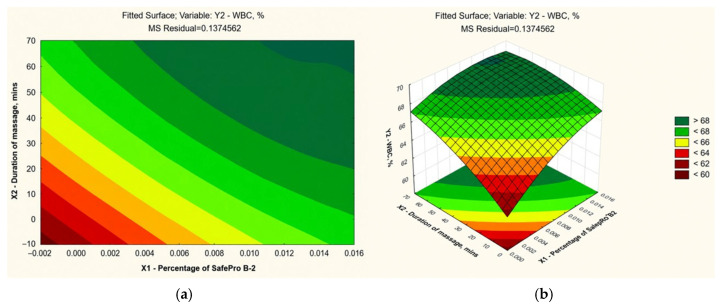
(**a**) Response surface for Y_2_ and (x_1_ and x_2_); (**b**) Contour lines for Y_2_ and (x_1_ and x_2_).

**Figure 4:**
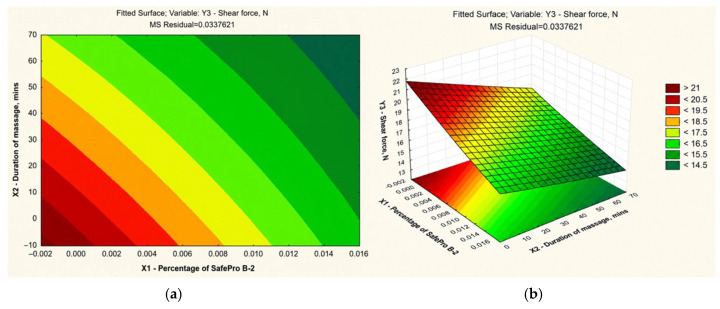
(**a**) Response surface for Y_3_ and (x_1_ and x_2_); (**b**) Contour lines for Y_3_ and (x_1_ and x_2_).

Figure 2, Figure 3 and Figure 4 show the response surface functions and contour lines (isolines) for the beef parameters Y_1_, Y_2_ and Y_3_ as a function of various combinations of the independent variables (x_1_ and x_2_).

Y_1_ (WHC, %): 58.3845 + 778.09·x_1_ + 0.0681·x_2_ − 0.819·x_1_·x_2_ −29432.91·x_1_^2^ − 0.000677·x_2_^2^; R^2^ = 0.999

For WHC (Y_1_), the response surface indicated a pronounced nonlinear relationship with the studied factors (Figure 2). Across the design space, the predicted WHC values increased from the lower-response region at reduced *L. sakei* content and shorter massage duration to a high-response region at intermediate-to-high L. *sakei* levels and medium massage duration. This trend is consistent with the experimental matrix, in which WHC varied from 62.55% to 64.59%. The steeper gradient of the surface along the X_1_ axis indicates that *L. sakei* content exerted a stronger effect on WHC than massage duration. However, due to the negative quadratic terms, the response did not continue to increase linearly at the upper factor levels and instead approached a plateau. The highest predicted WHC values were observed approximately in the range of 0.010–0.013% *L. sakei* and 40–50 min of massage, confirming the presence of a local optimum within the studied factor space.

The nonlinear WHC response may reflect the balance between protein hydration and structural loosening. Moderate *L. sakei* addition combined with tumbling may increase the accessibility of salt-soluble myofibrillar proteins and promote water immobilization within the protein matrix. Similar mechanisms have been described for meat systems in which water-holding capacity is controlled by myofibrillar spacing, protein charge, pH-dependent swelling and the integrity of the myofibrillar network [45]. Vacuum tumbling can further intensify this process by accelerating brine diffusion and improving the contact between curing ingredients and muscle proteins [46]. The plateau at higher factor levels suggests that the hydration capacity of the protein matrix approaches a technological limit within the studied range.

Y_2_ (WBC, %): 61.512 + 564.312·x_1_ + 0.118·x_2_ − 3.395·x_1_·x_2_ – 13,668.3544·x_1_^2^ − 0.000554·x_2_^2^; R^2^ = 0.987

For WBC (Y_2_), the response surface demonstrated a gradual and predominantly monotonic increase within the studied design space (Figure 3). The contour plot indicates that both increasing *L. sakei* content and extending massage duration contributed to higher WBC values. The smoother shape of the surface, compared with that obtained for Y_1_, suggests a weaker nonlinear component, which agrees with the regression results, where only the linear terms were statistically significant. The highest predicted WBC values were located in the upper region of the factor space, particularly at elevated *L. sakei* levels and longer massage duration. Therefore, unlike Y_1_, which exhibited a more distinct local optimum, Y_2_ was characterized mainly by the direct contribution of the two factors, with a tendency toward a plateau near the upper experimental limits.

The predominantly monotonic increase in WBC indicates that the ability of the meat system to bind water was mainly governed by cumulative brine distribution and protein hydration rather than by a sharply defined optimum. This agrees with reports that tumbling and marination improve water retention by increasing mass transfer, changing water mobility and promoting the swelling or extraction of myofibrillar proteins [47]. In the present study, the smoother WBC surface compared with WHC suggests that WBC was less sensitive to local structural limits and more dependent on the direct contribution of tumbling duration and microbial treatment within the selected factor range.

Y_3_ (shear force): 21.7815 − 422.7131·x_1_ − 0.0682·x_2_ + 2.3354·x_1_·x_2_ + 3769.62·x_1_^2^ + 0.00011·x_2_^2^; R^2^ = 0.996

For shear force (Y_3_), the response surface demonstrated a monotonic decrease within the experimental region (Figure 4). Both increasing *L. sakei* content and extending massage duration led to lower predicted shear force values, confirming the positive effect of these factors on meat tenderization. The steeper decline of the surface along the X_1_ axis suggests that *L. sakei* content exerted a stronger influence on Y_3_ than massage duration. At the same time, the response surface showed only limited curvature, which agrees with the regression results, where the linear terms and the interaction term were statistically significant, while the quadratic effects were not significant. The minimum predicted shear force values were located in the region of elevated *L. sakei* content and longer massage duration, i.e., close to the upper boundary of the factor space. Therefore, unlike Y_1_, which exhibited a local optimum, the behavior of Y_3_ was predominantly characterized by a direct decreasing response over the studied range, with an additional contribution from factor interaction.

The decreasing shear force surface has a clear biological interpretation—increasing microbial dose and tumbling duration progressively weakened the resistance of the muscle structure to cutting. In meat systems, shear force is determined by both myofibrillar integrity and connective tissue contribution. Therefore, any process that promotes myofibrillar fragmentation, protein swelling or fiber separation can reduce mechanical resistance [48]. Previous beef studies have shown that tumbling-curing can substantially reduce shear force and improve tenderness by altering the structural and functional properties of myofibrillar proteins [49]. The present response surface therefore reflects not only a geometric statistical trend but also a biologically plausible transition toward a more hydrated and less mechanically resistant muscle matrix.

A comparative analysis of the data (Table 7) obtained by calculation and experiment shows that the difference is no more than 0.5%.

The deviations between the calculated and experimental data are less than 0.1% for Y_1calc_ − Y_1exp_ and Y_3calc_ − Y_3exp_, and approximately 0.3% for Y_2calc_ − Y_2exp_, which confirms the accuracy and adequacy of the regression models developed.

**Figure 5:**
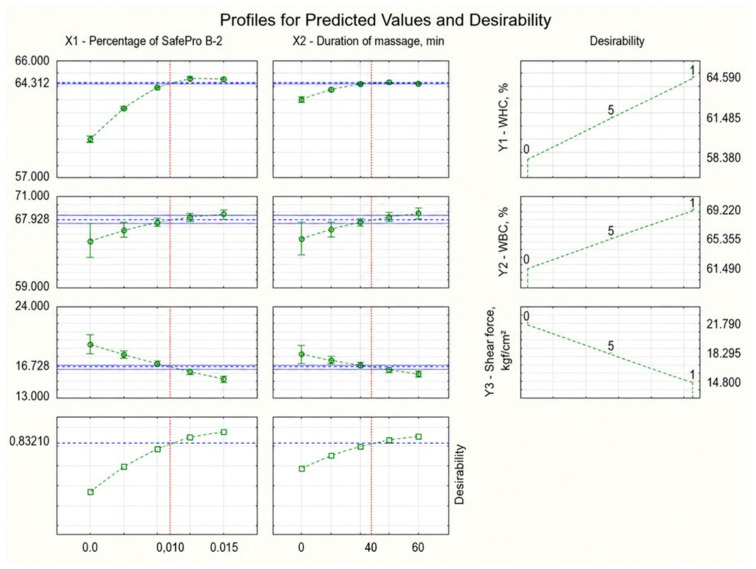
Predicted response and desirability profiles for multi-response optimization of *L. sakei* content and massage duration.

Because the response variables exhibited different optimization directions, the final process condition was selected using a multi-response desirability approach (Figure 5). In the optimization procedure, WHC (Y_1_) and WBC (Y_2_) were set to maximization, whereas shear force (Y_3_) was set to minimization. The overall optimum was therefore defined as the factor combination providing the best compromise.

The obtained desirability profile identified 0.010% *L. sakei* and 40 min of massage as the optimal processing conditions. This point corresponded to a balanced compromise among the three responses. WHC was close to its local maximum, WBC remained at a high level, and shear force was substantially reduced. Because this optimum coincided with one of the experimental runs, the predicted response values were compared directly with the observed data at the same design point and with the control sample (Table 8).

The optimization result should be interpreted as an internally verified multi-response compromise rather than as an externally validated optimum. The close agreement between predicted and experimental values at this point confirmed the internal consistency of the model within the original design space. However, this comparison should be considered internal verification only, because the selected point was included in the model-building dataset.

The selected optimum also reflects a trade-off between responses with different technological directions. Some factor combinations produced a lower shear force or a slightly higher WBC. For example, the combination of 0.015% *L. sakei* and 60 min tumbling resulted in the lowest shear force, while 0.010% *L. sakei* and 60 min tumbling gave the highest WBC. Therefore, the selected condition was not determined by shear force reduction alone, but by the simultaneous balance between high water retention, high water-binding capacity, and sufficient tenderization under a moderate microbial dose and tumbling duration. This interpretation is consistent with the desirability-function approach, where individual responses are transformed into a common desirability scale and optimized simultaneously, so the final solution represents the best overall compromise within the defined factor space rather than the absolute maximum or minimum of each response considered separately. In study, increasing treatment intensity further reduced mechanical resistance, whereas water-related responses approached a plateau within the investigated range.

Experimental and theoretical studies have demonstrated the validity of adding 0.01% *L. sakei* to the brine and that the optimal massaging time during the curing process is 40 min. To verify the reliability of the obtained results, studies were conducted on the physicochemical, structural-mechanical and microstructural parameters of the control and experimental meat samples with the optimal massaging time of 40 min with the addition of *L. sakei* in the range from 0.005% to 0.015%.

### 3.4. Physicochemical Properties

The mass fractions of the main components (protein, fat, ash, water) in raw meat samples show stable trends when *L. sakei* is added at levels of 0.005%, 0.01% and 0.015% combined with vacuum massaging (Table 9).

The protein content in all variants ranged from 22.07% to 22.43%, with no statistically significant differences (*p* > 0.05), indicating that the protein fraction remains stable regardless of the *L. sakei* concentration. This demonstrates that the introduction of the culture does not affect the overall protein content but rather modifies its functional state, which becomes evident in subsequent structural and textural changes.

A consistent reduction in fat content was observed, decreasing from 3.51% in the control to 2.87% at 0.015% *L. sakei*. In studies on feeding, reduced fat content has been linked to systemic metabolic effects [50]. In contrast, the present results suggest that the direct addition of *L. sakei* to raw meat systems followed by vacuum massaging may influence lipid distribution. This may indicate a local effect of the culture on lipid–protein interactions or phase redistribution in the meat matrix, which is a relatively understudied mechanism in raw, unfermented meat systems.

The ash content showed a moderate decrease (from 0.93% to 0.83%) at higher inclusion levels (0.010–0.015%). Unlike studies of formulated or emulsified products, where variations in ash content are often attributed to ingredient substitutions [51], the present data indicate that the addition of *L. sakei* in small doses, combined with vacuum mixing, can alter the mineral fraction. This may reflect a structural reorganization within the matrix that affects the retention or distribution of mineral components. In contrast, previous studies have reported simple dilution of the composition [52].

A gradual decrease in moisture content (from 73.13% to 70.83%) was observed as the concentration of *L. sakei* increased. Given that the additive was introduced in liquid form, this trend can be explained by a change in water-binding properties. The data indicate that *L. sakei*, when used in conjunction with tenderization methods, promotes the transition of free water into more tightly bound water, likely due to enhanced protein–protein interactions and matrix compaction [53].

It is important to note that this effect was demonstrated in this study using systems with raw meat treated with *L. sakei* and vacuum massaging, acting as a modulator of phase distribution (protein–lipid–water) [54]. The present results therefore provide new evidence that structural modification of the protein–water matrix can occur at early stages, prior to processing, and is strongly dose-dependent.

### 3.5. Amino Acid Content

The inclusion of *L. sakei* at different levels (0.005, 0.010, and 0.015%) and vacuum massaging noticeably influenced the amino acid profile of beef compared with the control sample (Table 10).

Most amino acids exhibited an increasing trend with the addition of *L. sakei*, particularly at higher inclusion levels (0.010–0.015%). The most pronounced increases were observed for glutamine, histidine, lysine, isoleucine, and several other amino acids, indicating a shift in the amino acid profile toward enhanced protein quality.

These results are consistent with data from international studies, which highlight the significant variability in the amino acid composition of meat depending on nutritional factors, rearing conditions and protein processing. Thus, an analysis of the profile of free and bound amino acids in various cuts of beef revealed that glutamine and lysine occupy the leading positions in terms of concentration among amino acids, and are also present in greater quantities than many other components, highlighting their importance for human nutrition [55].

In the study by Holló G. et al., variations in amino acid composition are attributed primarily to factors related to the animals: breed, feeding conditions, or post-slaughter processing [56]. The results of this study show that the direct introduction of *L. sakei* into raw meat systems can significantly alter the amino acid profile without changing the source of raw material.

The marked increase in glutamine and histidine, along with the substantial rise in isoleucine, suggests activation of proteolytic processes and selective accumulation of free amino acids. At the same time, the decrease in leucine alongside the increase in other branched-chain amino acids indicates a redistribution within the BCAA fraction rather than a uniform increase, as indicated by Duan Y. et al. [57].

These results provide new evidence that *L. sakei*, in combination with vacuum massaging, can influence protein transformation pathways at an early (preliminary) stage, leading to targeted changes in amino acid composition. This effect is likely dose-dependent and reflects not only general proteolysis but also selective modification of the amino acid balance in the meat matrix.

### 3.6. pH and Instrumental Color Characteristics

Table 11 shows the pH values and color characteristics (L*, a*, b*) of meat samples to which *L. sakei* was added at concentrations of 0.005%, 0.010% and 0.015% using vacuum massaging, compared with the control sample.

The control sample had a pH of 6.06 ± 0.10. The addition of *L. sakei* at concentrations of 0.005% and 0.010% significantly increased the pH to 6.42 ± 0.13 and 6.61, respectively (*p* < 0.05), whereas at 0.015%, a significant decrease to 5.62 ± 0.09 was observed. These results indicate a nonlinear effect of inoculation level on acid–base balance: low and moderate doses did not induce acidification, while the highest level led to pronounced pH reduction. This demonstrates that the metabolic activity of *L. sakei* in raw meat systems is strongly dose-dependent and can shift from neutral/alkalizing to acidifying with increasing inoculum concentration.

The L* value decreased progressively with increasing *L. sakei* concentration (from 50.29 ± 0.21 in the control to 39.58 ± 0.48 at 0.015%), indicating sample darkening. In the study by Pintado T. and Delgado-Pando G., color changes are mainly attributed to the intrinsic properties of the added ingredients [58]. In contrast, the present results show that the inclusion of microorganisms in combination with vacuum massaging, without the addition of exogenous pigments, can significantly alter lightness, indicating structural changes in the meat matrix.

The a* value increased from 15.01 ± 0.34 in the control group to 20.89 ± 0.53 at a concentration of 0.015%, indicating an intensification of the red hue. It is important to note that this effect was observed without the use of traditional color-stabilizing additives, as in the studies by Ruedt C. et al. [59]. This suggests that *L. sakei* may influence myoglobin stability through its metabolic activity and possesses a potential alternative color-enhancing mechanism based on microbiological modulation.

No significant differences in b* values (4.67–4.82) were observed, suggesting that the yellow color component is less sensitive to the inclusion of *L. sakei* and the mechanical processing method under the conditions studied.

In the study by Andrés-Bello A. et al., as in most published studies, fluctuations in pH and color are attributed primarily to individual ingredients or processing factors [60]. The present study demonstrates that direct inoculation of *L. sakei*, in combination with vacuum massaging, can significantly modulate both the acidity and color characteristics of raw meat systems. This result clarifies the functional role of microbial cultures, in combination with the vacuum massaging method, as active modifiers of physicochemical properties.

### 3.7. Texture Profile Analysis

The results of the textural profile analysis (TPA) for the control and experimental meat samples containing *L. sakei* in different levels are presented in Table 12. The addition of *L. sakei* and using vacuum massage had a statistically significant effect (*p* < 0.05) on all the textural parameters examined, in comparison with the control.

The hardness of the control sample was 12.95 ± 0.33 N. The addition of 0.005% *L. sakei* resulted in a significant increase in hardness to 13.58 ± 0.22 N (*p* < 0.05), while at 0.010%, the value (12.32 ± 0.14 N) did not differ from the control. In contrast, a pronounced decrease in hardness to 5.92 ± 0.35 N was observed at 0.015% *L. sakei* combined with vacuum massage, indicating substantial structural weakening. This effect can be attributed not only to microbial activity but also to the mechanical action of vacuum massage, which enhances protein extraction, disrupts muscle fiber integrity, and promotes redistribution of water and soluble proteins. The combined action of these factors results in a nonlinear, dose-dependent effect, where low concentrations enhance structural integrity, whereas high levels, especially under vacuum massage conditions, lead to matrix disruption.

Cohesiveness increased consistently with increasing *L. sakei* concentration, from 0.31 ± 0.06 in the control to 0.46 ± 0.04 at 0.015% (*p* < 0.05), indicating strengthening of internal structural interactions. The contribution of vacuum massage is particularly relevant here, as it facilitates protein solubilization (especially myofibrillar proteins), leading to improved binding between structural elements. Notably, the simultaneous increase in cohesiveness and decrease in hardness at 0.015% suggests the formation of a more plastic, less rigid structure, likely due to the combined effects of protein network rearrangement and mechanical tenderization, in contrast to studies reporting only matrix degradation [61].

Springiness reached its maximum at 0.005% (0.62 ± 0.25) and decreased to 0.41 ± 0.04 at 0.015%. This reduction reflects a diminished ability of the system to recover after deformation, which can be linked to both microbial-induced proteolysis and the physical disruption caused by vacuum massage. The latter reduces elastic resistance by weakening the organized muscle structure, contributing to a softer and less elastic texture.

Gumminess and chewiness decreased markedly with increasing *L. sakei* concentration: gumminess from 4.09 ± 0.27 N (control) to 2.69 ± 0.24 N (0.015%), and chewiness from 2.43 ± 0.21 to 1.11 ± 0.16 N·cm. These reductions are strongly influenced by vacuum massage, which enhances moisture retention and reduces structural rigidity, thereby lowering the energy required for mastication. Resilience also declined from 0.18 ± 0.05 to 0.12 ± 0.03, further confirming reduced structural recovery capacity, a typical consequence of both protein breakdown and mechanical processing. The results clearly show that *L. sakei* acts as an active modulator of textural properties in raw meat systems, whilst vacuum massage plays a synergistic role, enhancing structural changes.

In the studies by Xia Y. et al., as in many other studies by the authors, changes in texture are explained primarily by formulation components [62]. This work demonstrates that the combination of microbial inoculation and mechanical processing can significantly and selectively alter the mechanical properties of the protein matrix. It is important to note that a low concentration (0.005%) improves structural characteristics, whereas a higher concentration (0.015%), particularly under vacuum massage conditions, causes a transition to a softer and more deformable system. This allows the critical concentration threshold to be determined and highlights that the combined use of *L. sakei* and vacuum massage is an effective tool for targeted texture control without altering the basic formulation.

### 3.8. Microstructural Analysis

**Figure 6:**
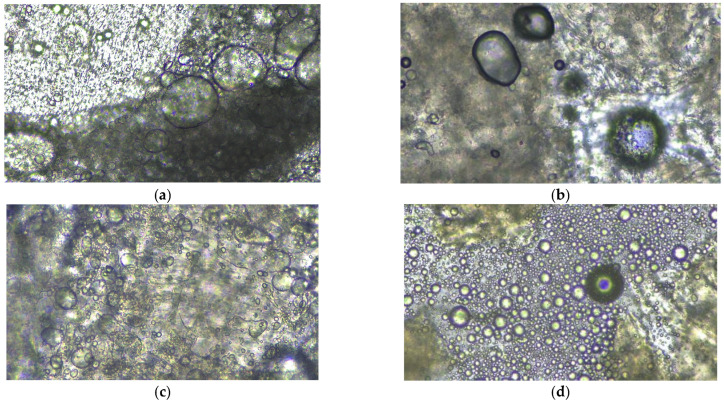
Images of microstructural analysis of control and experimental meat samples: (**a**) control, (**b**) SafePro-B2 (0.005%), (**c**) SafePro-B2 (0.010%), (**d**) SafePro-B2 (0.015%); control was a meat sample without *L. Sakei*.

Microstructural analysis revealed a clear concentration-dependent effect of *L. sakei* on muscle tissue organization, which is consistent with previously described patterns of protein–water interactions in processed meat systems (Figure 6). In addition, vacuum massage contributed significantly to the observed structural changes by enhancing mass transfer, promoting protein extraction, and inducing mechanical modification of muscle fibers, thereby intensifying the reorganization of the tissue matrix. The morphometric parameters of the microstructural elements are presented in Table 13.

The control sample exhibited a dense and well-organized structure, characterized by tightly packed muscle fibers with minimal inter-fiber space, indicating structural integrity.

The addition of 0.005% *L. sakei* induced moderate structural changes, primarily reflected in fiber swelling and a slight increase in inter-fiber distance. This effect is associated with initial protein hydration and partial matrix reorganization. Vacuum massage further contributed to these changes by facilitating water redistribution and increasing the accessibility of myofibrillar proteins, which was quantitatively confirmed by an increase in Feret’s diameter (up to 36.8 µm) and projection area (up to 810 µm^2^), without disruption of the overall structure [63].

With increasing concentration, structural modifications became more pronounced. At 0.015%, the samples showed a looser and more porous organization, with expanded inter-fiber spaces and formation of a more continuous matrix. Morphometric parameters increased significantly (Feret’s diameter 49.3 µm; projection area 1350 µm^2^), confirming the development of a more open and hydrated structure. This effect can be explained by the combined action of microbial activity and vacuum massage: while *L. sakei* contributes to biochemical modifications of the protein matrix (including proteolytic activity and changes in pH), vacuum massage intensifies structural disruption through mechanical forces, accelerating fiber separation and enhancing the formation of a protein-rich continuous phase. It is important to note that these changes were achieved without altering the composition of the raw materials, suggesting that *L. sakei*, in combination with vacuum massage, has a direct effect on the spatial organization of muscle fibers.

In the study by Absalimova M. et al., changes in microstructure are attributed almost exclusively to the added ingredients [64]. The results obtained here demonstrate that the synergy between microbial inoculation and mechanical processing can induce controlled restructuring of the meat matrix.

The observed gradual transition from a compact to a more open and hydrated structure reflects a shift in the balance between structural integrity and plasticity. This suggests that *L. sakei*, particularly in combination with vacuum massage, can be used as an effective tool for the targeted modification of meat microstructure and the development of products with specific textural properties.

## 4. Conclusions

This study demonstrated that the combined use of *L. sakei* in brine and vacuum massage has a significant effect on the physicochemical, structural–mechanical, and functional–technological properties of meat from culled cows. Response surface analysis confirmed a statistically significant effect of the factors on water-binding characteristics and shear force, with regression models demonstrating high adequacy (R^2^ = 0.91–0.96).

It was established that the combined treatment reduces shear force by 25–35% and increases water-holding capacity by 12–18% compared to the control. Optimal process parameters (10^7^ CFU/g; 40 min of massaging) provide the best combination of product softening and stability. Texture profile analysis revealed a statistically significant (*p* < 0.05) decrease in hardness, stickiness, and chewiness, accompanied by an increase in cohesiveness.

It has been shown that the observed effects are due to microstructural modification of muscle tissue and reorganization of the protein matrix, accompanied by moisture redistribution and increased accessibility of protein water-binding sites. Additionally, changes in amino acid composition were identified, reflecting the transformation of the state of protein components.

The scientific novelty of this study lies in the identification of a synergistic effect resulting from the combined use of *L. sakei* and vacuum massaging, as evidenced by improvements in structural and functional characteristics, as well as in the elucidation of the mechanism associated with microstructural rearrangement of the protein matrix. The developed model, based on the response surface method, allows for the prediction of product properties and the optimization of processing parameters for low-grade beef raw materials.

## Figures and Tables

**Table 1:** The levels of variation of independent variables.

Adjustable Parameters: Coded (natural)	Coded Levels			Range of Variation
	–1	0	+1	
X_1_—the amount of *L. sakei* (SafePro B-2), %	0.005	0.01	0.015	0.005
X_2_—Duration of massage, mins	20	40	60	20

**Table 2:** Planning matrix and results of FFE experiments.

Exp Number	Factors in a Dimensionless Coordinate System		Factors on a Real-World Scale		Output Parameter		
	Z_1_	Z_2_	X_1_	X_2_	Y_1_	Y_2_	Y_3_
			Percentage of *L. sakei* Added to the Raw Material Mass, %	Duration of Massage, Mins	WHC, %	WBC, %	Shear Force, N
1	−1	−1	0.005	20	62.55 ± 0.18	66.02 ± 0.28	18.50 ± 0.30
2	0	−1	0.01	20	64.16 ± 0.16	66.82 ± 0.24	17.30 ± 0.25
3	1	−1	0.015	20	64.28 ± 0.15	68.31 ± 0.22	15.60 ± 0.22
4	−1	0	0.005	40	63.05 ± 0.17	67.25 ± 0.24	17.80 ± 0.25
5	0	0	0.01	40	64.48 ± 0.15	68.29 ± 0.22	16.26 ± 0.20
6	1	0	0.015	40	64.59 ± 0.15	68.55 ± 0.21	15.10 ± 0.18
7	−1	1	0.005	60	62.92 ± 0.17	67.84 ± 0.23	16.80 ± 0.22
8	0	1	0.01	60	64.41 ± 0.16	69.22 ± 0.25	15.50 ± 0.20
9	1	1	0.015	60	64.34 ± 0.15	68.86 ± 0.22	14.80 ± 0.18
Control	-	-	0	0	58.38 ± 0.15	61.49 ± 0.30	21.79 ± 0.35

**Table 3:** Descriptive statistical characteristics of the response variables.

Statistical Characteristics	Symbol	Y_1_	Y_2_	Y_3_
Arithmetic mean	*M*	63.8644	67.9067	16.4067
Standard error	*m*	0.2628	0.3443	0.4259
Standard error, % of M	m, %	0.4115	0.5070	2.5958
Median	*med*	64.2800	68.2900	16.2600
Mode	*mod*	-	-	-
Standard deviation	*s*	0.7884	1.0328	1.2777
Sample variance	*s* ^2^	0.6215	1.0667	1.6324
Kurtosis	*E*	−1.2458	−0.7417	−1.1893
Skewness	*A*	−0.7227	−0.5732	0.3179
Range	*R*	2.0400	3.2000	3.7000
Minimum	*min*	62.5500	66.0200	14.8000
Maximum	*max*	64.5900	69.2200	18.5000
Coefficient of variation, %	*V*	1.2344	1.5209	7.7874

**Table 4:** Results of the regression analysis of the optimization control variables.

Factor	Regression Coefficient	Standard Error	Student’s *t* Test	*p*-Level of Significance	95% Confidence Interval	
					(Lower)	(Upper)
Y_1_—WHC, %						
—	58.3845	0.0371	1573.380	0.000000	58.3	58.5
x_1_	778.0886	16.3067	47.716	0.000001	732.8	823.4
$x_{1}^{2}$	−29432.9	89.567	−32.939	0.000005	−3191.9	−2695.0
x_2_	0.0681	0.0041	16.706	0.000075	0.1	0.1
$x_{2}^{2}$	−0.00068	0.0001	−12.123	0.000266	0.0	0.0
x_1_x_2_	−0.8199	0.1679	−4.884	0.008138	−1.3	−0.4
Y_2_—WBC, %						
—	61.512	0.360	170.8493	0.000000	60.5	62.51
x_1_	564.3122	158.215	3.5667	0.023444	125.0	1003.59
$x_{1}^{2}$	−13,668.4	866.828	−1.5765	0.190031	−3773.7	1040.95
x_2_	0.1184	0.040	2.9937	0.040192	0.0	0.23
$x_{2}^{2}$	−0.00055	0.001	−1.0229	0.364182	−0.0	0.00
x_1_x _2_	−3.3953	1.629	−2.0843	0.105504	−7.9	1.13
Y_3_—Shear force, N						
—	21.7815	0.178	122.0697	0.000000	21.29	22.28
x_1_	−422.713	78.412	−5.3909	0.005727	−640.42	−205.01
$x_{1}^{2}$	3769.62	429.778	0.8773	0.429850	−816.15	1569.39
x_2_	−0.0682	0.020	−3.4780	0.025399	−0.12	−0.01
$x_{2}^{2}$	0.000111	0.000	0.4118	0.701564	−0.00	0.00
x_1_x_2_	2.3354	0.807	2.8929	0.044433	0.09	4.58

**Table 5:** Results of the analysis of variance for the regression models obtained for the variables Y_1_, Y_2_ and Y_3_.

Source of Variability	Sum of Squares (SS)	Number of Degrees of Freedom df	Mean Square (MS)	F-Ratio	F-Score
Y_1_—WHC, %					
Regression (SS_R_)	32.0376	5	6.40752	4388.712	0.00000015
Error (SS_E_)	0.00584	3	0.00146		
Total SS (SS_T_)	32.04344	8			
Y_2_—WBC, %					
Regression (SS_R_)	45.03963	5	9.00793	65.53364	0.000629
Error (SS_E_)	0.54982	3	0.13746		
Total SS (SS_T_)	45.58945	8			
Y_3_—Shear force, N					
Regression (SS_R_)	39.0064	5	7.80128	231.0635	0.000052
Error (SS_E_)	0.13505	3	0.03376		
Total SS (SS_T_)	39.14145	8			

**Table 6:** Testing the adequacy and reliability of regression models for estimating the parameters Y_1_, Y_2_ and Y_3_.

Statistical Quality Indicators and Adequacy Criteria	Response		
	Y_1_—WHC, %	Y_2_—WBC, %	Y_3_—Shear Force, N
Multiple correlation, *R*	0.9999	0.994	0.9983
Coefficient of determination, *R*^2^	0.9998	0.9879	0.9966
Adjusted R-squared	0.9996	0.9729	0.9922
Standard error	0.0382	0.3708	0.1837
Number of degrees of independence, *df*: k_1_; *k*_2_	5; 3	5; 3	5; 3
Fisher’s criterion, *F*	4388.712	65.5336	231.0635
Significance, *F*	0.00000015	0.000629	0.000052
The Durbin–Watson criterion, *d*	1.7946	1.7032	1.8284

Note: k_1_ and k_2_ are the numbers of degrees of freedom for the numerator and denominator, respectively.

**Table 7:** Comparative evaluation of Y_1_, Y_2_ and Y_3_, obtained experimentally (Yi_exp_) and by calculation (Yi_calc_).

№ exp	Y_1calc_	Y_1exp_	Y_2calc_	Y_2exp_	Y_3calc_	Y_3exp_
1	62.55	62.55	65.79	66.02	18.68	18.5
2	64.15	64.16	67.25	66.82	17.08	17.3
3	64.28	64.28	68.02	68.31	15.67	15.6
4	63.02	63.05	67.15	67.25	17.68	17.8
5	64.54	64.48	68.26	68.29	16.31	16.26
6	64.58	64.59	68.70	68.55	15.14	15.1
7	62.94	62.92	68.06	67.84	16.77	16.8
8	64.38	64.41	68.84	69.22	15.64	15.5
9	64.35	64.34	68.93	68.86	14.69	14.8

Standard deviations: ΔY_1_ ≈ 0.05%; ΔY_2_ ≈ 0.26%; ΔY_3_ ≈ 0.09%.

**Table 8:** Comparison of predicted optimum, experimental optimum, and control values.

Parameter	Predicted Values	Experimental Values	Control
WHC, %	64.54	64.48 ± 0.15	58.38 ± 0.15
WBC, %	68.26	68.29 ± 0.22	61.49 ± 0.30
Shear force, N	16.31	16.26 ± 0.20	21.79 ± 0.35

**Table 9:** Physicochemical properties of the test samples.

Chemical Content	Control	SafePro B-2 (0.005%)	SafePro B-2 (0.010%)	SafePro B-2 (0.015%)
Protein, %	22.07 ± 0.10 ^a^	22.34 ± 0.11 ^a^	22.26 ± 0.9 ^a^	22.43 ± 0.14 ^a^
Fat, %	3.51 ± 0.25 ^a^	3.32 ± 0.29 ^a^	2.98 ± 0.31 ^b^	2.87 ± 0.32 ^b^
Ash, %	0.93 ± 0.22 ^a^	0.85 ± 0.12 ^b^	0.87 ± 0.25 ^b^	0.83 ± 0.16 ^bc^
Water, %	73.13 ± 0.10 ^a^	71.12 ± 0.27 ^b^	71.25 ± 0.31 ^b^	70.83 ± 0.35 ^c^

Control was a raw meat without L. Sakei; values are shown as mean ± SD; letters (a–c) represent significant differences at *p* < 0.05.

**Table 10:** Amino acid content.

Beef	Control	SafePro B-2 (0.005%)	SafePro B-2 (0.010%)	SafePro B-2 (0.015%)
Essential amino acids				
Asparagine	83.47 ± 2.13	92,32 ± 1.45	84,12 ± 1.32	56.32 ± 0.63
Serine	45.57 ± 1.97	46.25 ± 0.96	45.98 ± 0.68	45.64 ± 0.57
Glutamine	37.66 ± 1.39	52.34 ± 0.45	69.21 ± 1.52	73.36 ± 2.00
Glycine	10.61 ± 0.65	15.54 ± 0.57	17.21 ± 0.45	20.35 ± 0.68
Histidine	97.16 ± 2.17	105.25 ± 2.62	120.17 ± 2.24	137.27 ± 2.97
Tryptophan	10.17 ± 0.70	12.14 ± 0.52	12.85 ± 0.60	15.53 ± 0.70
Arginine	123.36 ± 2.98	125.25 ± 3.65	135.65 ± 2.85	134.72 ± 5.56
Alanine	83.80 ± 2.62	85.70 ± 2.14	86.12 ± 3.13	99.52 ± 2.81
Proline	37.09 ± 1.72	38.12 ± 1.61	41.45 ± 1.47	44.63 ± 1.82
Cysteine	2.17 ± 0.23	2.41 ± 0.30	2.49 ± 0.21	2.55 ± 0.26
Non-essential amino acids				
Threonine	40.50 ± 1.50	40.61 ± 0.95	41.98 ± 0.12	40.60 ± 0.17
Tyrosine	35.47 ± 1.43	35.85 ± 0.98	38.56 ± 1.12	40.18 ± 0.81
Valine	22.42 ± 1.14	24.54 ± 0.56	26.21 ± 0.96	26.43 ± 0.89
Methionine	21.35 ± 1.08	21.55 ± 0.85	23.01 ± 1.12	24.67 ± 0.77
Lysine	106.10 ± 2.93	105.23 ± 2.45	110.54 ± 2.78	118.11 ± 2.32
Isoleucine	23.98 ± 1.16	30.65 ± 1.23	43.23 ± 1.12	64.27 ± 1.57
Leucine	52.68 ± 1.89	45.32 ± 1.99	40.04 ± 1.78	28.94 ± 0.96
Phenylalanine	20.10 ± 1.13	21.65 ± 1.45	20.05 ± 1.08	22.92 ± 0.76

**Table 11:** pH and color of meat samples.

Trait Sample	Control	SafePro B-2 (0.005%)	SafePro B-2 (0.010%)	SafePro B-2 (0.015%)
pH	6.06 ± 0.10 ^b^	6.42 ± 0.13 ^a^	6.61 ± 0.21 ^a^	5.62 ± 0.09 ^c^
L*	50.29 ± 0.2 ^a^	48.65 ± 0.2 ^b^	43.76 ± 0.56 ^c^	39.58 ± 0.48 ^d^
a*	15.01 ± 0.34 ^c^	17.54 ± 0.76 ^b^	20.54 ± 0.26 ^a^	20.89 ± 0.53 ^a^
b*	4.72 ± 0.23 ^b^	4.82 ± 0.46 ^a^	4.67 ± 0.59 ^bc^	4.79 ± 0.43 ^b^

Values are presented as mean ± standard deviation (*n* = 3). Different superscript letters within the same row indicate significant differences (*p* < 0.05).

**Table 12:** Structural and mechanical properties of the control and test samples.

Parameters	Control	SafePro B-2 (0.005%)	SafePro B-2 (0.010%)	SafePro B-2 (0.015%)
Hardness, N	12.95 ± 0.33 ^b^	13.58 ± 0.22 ^a^	12.32 ± 0.14 ^b^	5.92 ± 0.35 ^c^
Cohesiveness, -	0.31 ± 0.06 ^d^	0.40 ± 0.01 ^bc^	0.42 ± 0.02 ^b^	0.46 ± 0.04 ^a^
Springiness, -	0.59 ± 0.06 ^b^	0.62 ± 0.05 ^a^	0.53 ± 0.06 ^bc^	0.41 ± 0.04 ^d^
Gumminess, N	4.09 ± 0.27 ^a^	3.25 ± 0.11 ^b^	3.62 ± 0.18 ^b^	2.69 ± 0.24 ^c^
Chewiness, N·cm	2.43 ± 0.21 ^a^	2.02 ± 0.25 ^ab^	1.85 ± 0.28 ^c^	1.11 ± 0.20 ^cd^
Resilience, -	0.18 ± 0.05 ^a^	0.15 ± 0.04 ^b^	0.12 ± 0.03 ^c^	0.12 ± 0.03 ^c^

An average sample from each group (replicate) was tested, with three replicates per analysis. Control was a meat sample without *L. Sakei*; letters (a–d) represent significant differences at *p* < 0.05.

**Table 13:** Morphometric characteristics of the structure of meat samples.

Treatment	Feret’s Diameter (μm)	Equivalent Diameter (μm)	Projection Area (μm^2^)
Control	32.4 ± 4.1 ^d^	28.7 ± 3.6 ^d^	650 ± 85 ^d^
SafePro B-2 (0.005%)	36.8 ± 5.2 ^c^	32.1 ± 4.4 ^c^	810 ± 110 ^c^
SafePro B-2 (0.010%)	42.6 ± 6.8 ^b^	37.9 ± 5.9 ^b^	1020 ± 160 ^b^
SafePro B-2 (0.015%)	49.3 ± 9.5 ^a^	44.7 ± 8.2 ^a^	1350 ± 240 ^a^

Values are shown as mean ± SD. Different letters (a–d) represent significant differences at *p* < 0.05.

## Data Availability

The original contributions presented in the study are included in the article. Further inquiries can be directed to the corresponding authors.

## Abbreviations

The following abbreviations are used in this manuscript:

WHC	Water-holding capacity
WBC	Water-binding capacity
GRAS	Generally recognized as safe
LAB	Lactic acid bacteria
TR CU	Technical Regulation of Customs Union
SS	Software suite
HPLC	High-performance liquid chromatography
FFE	Full factorial experiment
RSM	Response surface method
BCAA	Branched-chain amino acids
PSE	Pale, Soft, Exudative
DFD	Dark, Firm, Dry
